# Neural correlates of working memory training in HIV patients: study protocol for a randomized controlled trial

**DOI:** 10.1186/s13063-016-1160-4

**Published:** 2016-02-02

**Authors:** L Chang, GC Løhaugen, V Douet, EN Miller, J Skranes, T Ernst

**Affiliations:** Department of Medicine, John A. Burns School of Medicine, The Queen’s Medical Center, University of Hawaii at Manoa, 1356 Lusitana Street, 7th Floor UH Tower, Honolulu, HI USA; Department of Pediatrics, Sørlandet Hospital, Arendal, Norway; Department of Laboratory Medicine, Children’s and Women’s Health, Norwegian University of Science and Technology, Trondheim, Norway; UCLA Psychiatry and Biobehavioral Sciences, Los Angeles, CA USA

**Keywords:** Cogmed®, Computerized training, fMRI, HIV, Working memory

## Abstract

**Background:**

Potent combined antiretroviral therapy decreased the incidence and severity of HIV-associated neurocognitive disorders (HAND); however, no specific effective pharmacotherapy exists for HAND. Patients with HIV commonly have deficits in working memory and attention, which may negatively impact many other cognitive domains, leading to HAND. Since HAND may lead to loss of independence in activities of daily living and negative emotional well-being, and incur a high economic burden, effective treatments for HAND are urgently needed. This study aims to determine whether adaptive working memory training might improve cognitive functions and neural network efficiency and possibly decrease neuroinflammation. This study also aims to assess whether subjects with the *LMX1A*-rs4657412 TT(AA) genotype show greater training effects from working memory training than TC(AG) or CC(GG)-carriers.

**Methods/Design:**

60 HIV-infected and 60 seronegative control participants will be randomized to a double-blind active-controlled study, using adaptive versus non-adaptive Cogmed Working Memory Training® (CWMT), 20–25 sessions over 5–8 weeks. Each subject will be assessed with near- and far-transfer cognitive tasks, self-reported mood and executive function questionnaires, and blood-oxygenation level-dependent functional MRI during working memory (*n*-back) and visual attention (ball tracking) tasks, at baseline, 1-month, and 6-months after CWMT. Furthermore, genotyping for *LMX1A-*rs4657412 will be performed to identify whether subjects with the TT(AA)-genotype show greater gain or neural efficiency after CWMT than those with other genotypes. Lastly, cerebrospinal fluid will be obtained before and after CWMT to explore changes in levels of inflammatory proteins (cytokines and chemokines) and monoamines.

**Discussion:**

Improving working memory in HIV patients, using CWMT, might slow the progression or delay the onset of HAND. Observation of decreased brain activation or normalized neural networks, using fMRI, after CWMT would lead to a better understanding of how neural networks are modulated by CWMT. Moreover, validating the greater training gain in subjects with the *LMX1A*-TT(AA) genotype could lead to a personalized approach for future working memory training studies. Demonstrating and understanding the neural correlates of the efficacy of CWMT in HIV patients could lead to a safe adjunctive therapy for HAND, and possibly other brain disorders.

**Trial registration:**

ClinicalTrial.gov, NCT02602418.

## Background

The incidence of HIV-associated dementia has declined dramatically since the introduction of potent combined antiretroviral therapy; however, milder forms of HIV-associated neurocognitive disorders (HAND) persist. Some 30 to 50 % of HIV-infected individuals have HAND, which is more prevalent in those older than 50 years [1, 2]. Other co-morbidities, such as substance abuse (e.g., alcohol, cannabis, or psychostimulants) and hepatitis C infection, further exacerbate or hasten the development of HAND, partly owing to poorer medication adherence compared with those without HAND [3, 4]. With an increasing proportion of HIV-infected individuals at risk of HAND, which leads to both functional and occupational disabilities, the development of effective treatments or adjunctive therapy for these patients is critical.

The current therapy for HIV infection leaves a ‘therapeutic gap’ for HAND [1, 2]. Combined antiretroviral therapy is effective at suppressing the viral burden but cannot reverse HAND completely or prevent the development of HAND, even in those with long-standing aviremia [5]. In addition, antiretroviral drugs may have adverse or neurotoxic effects on the brain [6–8]. A number of pharmacological trials, including trials of minocycline [9], CEP-1347 [10], memantine [11], lithium [12], peptide T [13], and selegiline [14–16], were conducted with no clear efficacy for cognitive improvements, although some of these studies demonstrated positive effects on neuroimaging measures in human beings or immunohistochemistry in animal models. Therefore, no evidence-based treatment is available to improve the cognitive function of patients with HAND. In addition, neuropathological, neuroimaging, and cerebrospinal fluid studies consistently showed ongoing aberrant neuroinflammation, with persistent glial activation [17], and lesser dopamine transporters [18, 19]; both may contribute to brain injury and HAND in HIV patients. Hence, effective treatment for HAND might require a multimodal approach.

Another important reason to develop an effective treatment is that HAND leads to higher healthcare costs. For instance, a study of patients with HIV or AIDS in a community-based clinic in Canada found that the care for those with a neuropsychiatric disorder was more costly, both immediately before and after the diagnosis, regardless of the specific neuropsychiatric diagnosis [20]. Likewise, an Australian study showed that with the predicted doubling of HIV infection in men and tripling in women over the next two decades, the cost of care could nearly double at the current 7 % prevalence of HIV-associated dementia, primarily for residential care [21]. Another study from Sweden on the pharmacoeconomics of mild cognitive impairment showed that postponing the development of dementia even for a few years after diagnosis of mild cognitive impairment would result in cost savings of approximately $5,300 per person [22]. Therefore, an effective treatment for HAND or to delay the onset of HAND, even in a subset of HIV-infected individuals, might lead to significant cost savings.

The major cognitive domains affected in HAND are attention and working memory [23, 24], in both visual and verbal domains [25–28]. Working memory is defined as the cognitive skill to retain and manipulate information ‘online’ over short periods of time. Working memory is thus necessary for concentration and maintaining awareness and is crucial for learning and executive functions, such as reasoning and planning. Working memory is also needed in daily life in all settings. Hence, deficits in working memory may negatively affect many other cognitive domains. While the introduction of combined antiretroviral therapy led to generally improved neuropsychological function, working memory dysfunction was not improved or was even exacerbated with ongoing infection [1, 29]. Working memory dysfunction in HIV has major consequences, such as being a strong predictor of unemployment and dependence in activities of daily living [30], self-reported cognitive complaints [31], and poorer medication adherence [32, 33]. Therefore, enhancing working memory ability and delaying progression of working memory decline should benefit HIV-infected individuals.

Importantly, working memory skills can be trained, for instance, using Cogmed Working Memory Training® (CWMT), a computer-based adaptive working memory training program that is effective for improving cognition. The efficacy of CWMT was first demonstrated in children with attention deficit hyperactivity disorders [34], who showed better non-verbal and verbal working memory performance and improved response inhibition and reasoning, as well as reduced parent-rated inattentive symptoms of attention deficit hyperactivity disorders [35]. Patients with traumatic brain injury also had improved functioning in daily life activities, which was maintained even 6 months after CWMT [36]. Other populations who benefitted from CWMT included stroke victims [37], adolescents born preterm with extremely low birth weight [38], children with cochlear implants [39], and healthy older persons [40]. However, this remarkably safe and innovative intervention has not been evaluated as an adjunctive therapy for HAND.

To our knowledge, no study has investigated the neural correlates of working memory training in HIV patients, or whether neural networks might re-organize or show corresponding improved neural efficiency. Working memory deficits in HIV patients probably involve injuries to the frontostriatal circuits, which are brain regions that have been shown in neuropathological studies [41] to be preferentially affected in HIV patients. Working memory deficits have been observed in encoding [23], reaction times [42], and simultaneous short-term storage and processing [43], suggesting subcortical deficits as well as abnormalities of the prefrontal cortex [26]. The brain is highly ‘plastic’ in that brain activities and network connections can change quickly and that changes can last for a long time after a task is practiced, as found in tasks involving visual attention [44], object naming [45], motor learning [46, 47], and working memory [48–50]. Blood-oxygenation level-dependent functional magnetic resonance imaging (BOLD-fMRI) studies following 5-weeks of adaptive CWMT showed increased training-related activity in the medial frontal gyrus and parietal cortices for the trained tasks [51, 52] and in the the inferior frontal gyrus for the untrained tasks with varied working memory load [52]. However, compared with fixed low-level training (active control condition), older adults who received adaptive working memory training showed decreased brain activation in frontal, parietal, and temporal cortices but increased activity in the caudate and thalamus [53]. Transfer of performance improvement (gain) to untrained tasks (transfer of gain) requires overlapping networks and brain regions common to both tasks, especially the striatum [54]. Our proposed fMRI studies will further assess brain changes associated with improvements on working memory and other cognitive tasks.

Recent data also indicate that individuals with different genotypes for dopamine-related genes may have different training gain, such as polymorphisms in *LMX1A* [55] and *catechol-O-methyltransferase Val158Met* [56]. This study will focus on *LMX1A*, which encodes for the protein LIM homeobox transcription factor 1 alpha, a positive regulator of insulin gene transcription, but is also involved in the proliferation, differentiation, and maintenance of dopamine-producing neurons in the midbrain [57–59]. Polymorphisms in *LMX1A* were associated with dopaminergic disorders, such as Parkinson’s disease [60] and schizophrenia [61], and, recently, with the effects of working memory training [55]. Specifically, subjects with TT(AA) alleles at the *LMX1A*-rs4657412 had greater training-related gains in verbal working memory than those with heterozygous TC(AG) or CC(GG) alleles [55]. Since HIV patients commonly showed dopaminergic deficits, with decreased dopamine transporters [18, 19] and decreased cerebrospinal fluid dopamine and dopamine metabolites [62], we will explore whether genetic variations of *LMX1A*-rs4657412 modulates working memory training effects and cerebrospinal fluid monoamines and their metabolites in our subjects.

Based on this prior knowledge, the current study has three major aims and one exploratory aim.

- Aim 1: To perform a double-blind active-controlled study on the effectiveness of CWMT (25 sessions over 5–8 weeks) in HIV-infected and seronegative subjects, with or without cognitive deficits, and assess whether the training will improve cognitive functioning (on working memory and some non-trained tasks that require working memory) at 1 month (to assess gain) and 6 months (to assess maintenance of gain) after the training.
- Aim 2: To assess the neural correlates of working memory training, and determine whether brain activation will improve, at 1 month and 6 -months after the training, as assessed by functional MRI (fMRI) during working memory and attention tasks.
- Aim 3: Since individuals with the TT(AA) genotype for the single nucleotide polymorphism *LMX1A*-rs4657412 had greater magnitude of training-related gains in verbal working memory [55], we will assess our subjects for the allelic variations on training results (gain and maintenance).

### Exploratory aim

We will explore changes in neuroinflammatory markers, and monoamine and their metabolites in the cerebrospinal fluid before and at least 1 month after CWMT, and evaluate the relationships between cerebrospinal fluid markers, cognitive performance, and BOLD-fMRI signals.

## Methods/design

**Fig. 1:**
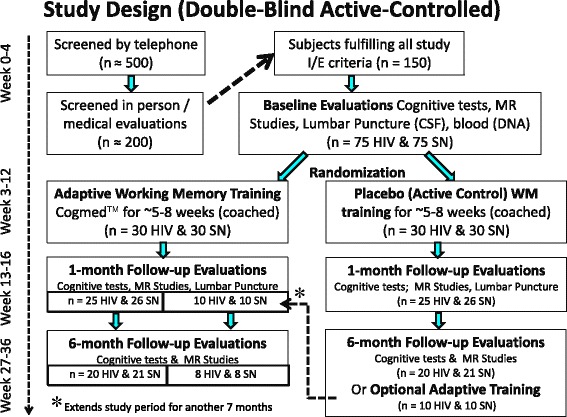
Study design. We plan to screen ≈ 500 individuals initially by telephone and 200 of these potentially eligible participants in person, to identify ≈ 150 subjects who will fulfill our study criteria, and complete the baseline evaluation. We expect that only ≈ 120 (60 HIV and 60 seronegative) will follow through and be randomized for the training. We also expect that only 85 % (*n* = 102, 50 HIV, and 52 seronegative) will return for the 1 month follow-up and only 80 % of these (*n* = 86, 40 HIV, and 42 seronegative) will return to complete the 6-month follow-up evaluations. Furthermore, we expect that ≈ 20 participants who completed the ‘active control’ training will want to continue with the ‘adaptive training’. CSF, cerebrospinal fluid; I/E, inclusion or exclusion; MR, magnetic resonance; SN, seronegative; WM, working memory.

The proposed study uses a 2 × 2 repeated-measures analysis of covariance (ANCOVA) design to evaluate the effects of 5 weeks of CWMT, adaptive versus ‘active control (fixed low level)’ training, as assessed by the improvement index (generated from task performances within the CWMT), neurocognitive testing, BOLD-fMRI measurements, cerebrospinal fluid neuroinflammatory proteins, monoamine, and metabolite levels. We propose to enroll 60 HIV-infected individuals and 60 seronegative healthy participants, with 30 adaptive and 30 active-control-training subjects randomized in each group (Fig. 1). HIV-positive and seronegative subject groups will be matched by age, sex, education, socioeconomic status, and cognitive status (matched by global cognitive score from the seven cognitive domains tested), as well as other co-variates (e.g., nicotine smoking and other substances abused). Based on the initial screening evaluations, subjects who fulfill all study criteria will be scheduled for a baseline neuropsychological evaluation, MRI-fMRI scans, and a lumbar puncture.

### Primary and secondary outcome measures

The main goal of this study is to examine the effectiveness of intensive adaptive working memory training for the treatment of HAND. The primary outcome measures will be the improvement index on CWMT, improvement on non-trained near-transfer working memory tasks, and changes on brain activation assessed with BOLD-fMRI during working memory and attention tasks. Secondary contrasts will evaluate genotype effects (*LMX1A*, TT(AA) versus TC(AG) or CC(GG)) on cognitive improvements and BOLD-fMRI. We will also assess CWMT-related improvements on self-reported depressive and various psychopathological symptoms, using the Center for Epidemiological Studies–Depression Scale Revised and Symptom Checklist–90 revised items, and on self-rated functionality, using the Independent Activity of Daily Living assessment and the Behavior Rating Inventory of Executive Function–Adult Version.

### Participants

Subjects will be recruited via flyers, referrals from local HIV clinics, by word of mouth, or from ongoing studies. Potential research subjects will be screened by telephone or on-site, before being scheduled for a detailed out-patient evaluation at the UH-QMC MR Research Center at the Queen’s Medical Center in Honolulu, Hawaii.

In our pilot studies, we observed effect sizes from 0.7 to 1.2 for pre versus post CWMT in HIV-positive subjects in four of the working memory tests. We will need to enroll ≈ 17 HIV-positive subjects to detect an effect size of 0.7 for 80 % power (two-sided test). Effect sizes for BOLD signal change (pre versus post adaptive CWMT (0 and 1-back)) were 0.58–1.5 in the pilot HIV-positive subjects; at least 26 HIV-positive subjects are needed to detect an effect size of 0.58 for 80 % power (two-sided test). Since we encountered an ≈ 15 % (13 % in HIV and 20 % in the seronegative group) attrition rate in our pilot study, we propose to enroll 30 HIV and 30 seronegative subjects per training group to ensure adequate power, for a total of 120 subjects (60 HIV, 60 seronegative). Based on our experience, we will have to screen ≈ 500 individuals initially by telephone and 200 of these potentially eligible participants in person to have ≈ 150 subjects who will fulfill our study criteria, and complete the baseline evaluation. We expect that only ≈ 120 subjects (60 HIV and 60 seronegative) will follow through and be randomized for the training. We also expect that only 85 % (*n* = 102, 50 HIV and 52 seronegative) will return for the 1 month follow-up and only 80 % of these (*n* = 86, 40 HIV and 42 seronegative) will return to complete the 6-month follow-up evaluations. Furthermore, we expect that ≈ 20 participants who completed the ‘active control’ training will want to continue with the ‘adaptive training’ (Fig. 1).

HIV-infected participants must meet the following inclusion criteria: (1) men or women of any ethnicity, ages ≥ 18 years and able to provide informed consent; (2) HIV seropositive (with documentation from medical records); (3) stable on an antiretroviral regimen for 6 months or will remain without antiretroviral treatment during the duration of the CWMT study.

Seronegative healthy participants must meet the following inclusion criteria: (1) men or women of any ethnicity, ages ≥ 18 years and able to give informed consent; (2) seronegative for HIV (to be confirmed by an HIV ELISA blood test if screened positive with ClearView® COMPLETE HIV-1/2 test).

Exclusion criteria for all subjects are: (1) history of co-morbid psychiatric illness that might confound the analysis of the study (e.g., schizophrenia, obsessive-compulsive disorder, major depression, or bipolar disorder); (2) confounding neurological disorder (e.g., multiple sclerosis, Parkinson’s disease, non-HIV brain infections, neoplasms, cerebral palsy, or significant head trauma with loss of consciousness >30 minutes); (3) significantly abnormal screening laboratory tests (>2 standard deviations) that might indicate a chronic medical condition (e.g., diabetes, severe cardiac, renal or liver disorders) that might affect brain function; (4) medications that could significantly alter functional brain imaging studies; (5) current or history of drug dependence within the previous 2 years, including amphetamines, cocaine, alcohol, and opiates, according to DSM-V dependency criteria (past casual or recreational usage will not be excluded); (6) positive urine toxicology screen (methamphetamine, amphetamine, cocaine, marijuana, benzodiazepine, barbiturates, and opiates except for false positive tests due to medications) on day of neuropsychological testing or fMRI studies; (7) pregnancy (to be verified by urine pregnancy test in women of child-bearing age); (8) inability to read at an 8th-grade level (to be verified by the Wechsler Test of Adult Reading); (9) other contraindications for MRI studies, such as metallic or electronic implants in the body (e.g., pacemaker, surgical clips, or pumps), or severe claustrophobia.

### Baseline assessments

We will first explain the study to each subject and obtain oral and written consent. The study design, experiments, and consent forms were approved by the institutional review board of the University of Hawaii at Manoa (CHS#20523). A detailed medical and drug use history and physical and neuropsychiatric evaluations will be performed, along with screening blood tests to assess general health, including complete blood cell count, chemistry panel, thyroid function tests, hepatitis C and HIV serology. An electrocardiogram will be obtained to screen for significant cardiac abnormalities; subjects will be referred for further cardiac evaluations should significant abnormalities be found. Psychiatric and quality-of-life evaluations will be additionally performed through a battery of questionnaires and self-reported assessments; some will be repeated after training (Table 1).

**Table 1:** Questionnaires and interviews

	Outcome measures	Baseline	1 month after training	6 months after training
Hollingshead Four Factor Index [90]	Socioeconomic status; combination of race, ethnicity, occupational status and education level	✓		
Psychiatric assessments				
Symptom Checklist–90–revised	Psychological and emotional distress	✓	✓	✓
Center for Epidemiological Studies–Depression Scale [91]	Depressive symptoms and to rule out possible major depression	✓	✓	✓
Substance use assessments				
Substance Abuse Subtle Screening Inventory-3 [92]	Identification of individuals with a high probability of having a substance use disorder	✓	✓	✓
Urine toxicology	Substance usage	✓	✓	✓
Quality-of-life assessments				
Independent Activity of Daily Living [93]	Ability to carry out daily functions	✓	✓	✓
Behavior Rating Inventory of Executive Function–Adult Version	Executive function	✓	✓	✓
Pittsburgh Sleep Quality Index [94], Epworth Sleepiness Scale [95]	General level of daytime sleepiness	✓	✓	✓
PROMIS®	Global health status and pain intensity, interference and behavior	✓	✓	✓
Medical Outcomes Study HIV Health Survey [96]	Health status for HIV-positive patients	✓	✓	✓
World Health Organization Quality of Life [97]	Well-being and quality of life	✓	✓	✓
Biospecimens				
Blood	DNA and genotyping	✓		
Blood and serum	Cytokines and monoamines and neurometabolite measurements	✓	✓	✓
Cerebrospinal fluid	Cytokines monoamines and neurometabolite measurements	✓	✓	

Subjects with significant psychiatric symptoms will be further assessed by a qualified physician, to determine whether psychiatric referral is necessary. Those with suicidal thoughts or acute intoxication will be sent to the psychiatric emergency department. Subjects with significant medical problems, screening blood test abnormalities, or electrocardiographic abnormalities will be referred to their primary care physicians, other clinics or the emergency department as needed.

### Neuropsychological evaluation

The 2 hour long neuropsychological test battery is designed to detect deficits in (1) working memory and attention, which implicate networks in frontal and parietal regions; (2) decision making and inhibition, which involve primarily the anterior cingulate and orbitofrontal cortices; and (3) motor and psychomotor speed, which are associated with alterations in the basal ganglia and cerebellum.

1. Diagnosis of HAND or HAND-equivalent at baseline will be determined using z-scores from seven cognitive domains, adjusted for age and education, and derived from our existing normative database (including ≈ 450 healthy seronegative controls tested with the same protocol). HAND classification will be made according to the Updated Research Nosology for HAND [63] by physicians for all subjects. The HIV-positive and seronegative control groups will be matched by their cognitive status (i.e., global z-score) (Table 2).

**Table 2:** Neuropsychological assessments (non-trained working memory tests)

	Baseline	1 month after training	6 months after training
Attention, working memory			
Paced Auditory Serial Addition Test	✓		
Wechsler Adult Intelligence Scale IV (Digit Span, Letter Number Sequencing, Arithmetic, and Digit-Symbol Coding)	✓	✓	✓
California Computerized Assessment Package	✓	✓	✓
Delis–Kaplan Executive Function System	✓	✓	✓
Abstraction, executive functions			
Stroop Color Interference Test	✓		
Trail Making Test Part B	✓		
Fluency			
Controlled Oral Word Association Test (FAS)	✓		
Design Fluency Tests (Ruff Figural Fluency)	✓		
Memory			
Rey Auditory Verbal Learning Test Delayed Recall	✓	✓	✓
Rey Osterrieth Complex Figure Test	✓	✓	✓
Speed of information processing, psychomotor speed			
Symbol Digit Modalities Test	✓		
Trail Making Test Part A	✓		
Motor skills			
Grooved pegboard test (dominant and non-dominant hand)	✓		
Verbal, language			
Wechsler Adult Intelligence Scale, fourth edition, verbal comprehension index	✓		
Wechsler Test of Adult Reading	✓		
Wechsler Adult Intelligence Scale	✓		
Wechsler Memory Scale IV			
Spatial span	✓	✓	✓
Visual, verbal immediate and long-term memory	✓	✓	✓
Mental control	✓	✓	✓
2. General intelligence: the Wechsler Test of Adult Reading will provide an estimate of premorbid intellectual functioning during our screening procedure to ensure that the subjects have a verbal intelligence quotient > 80 and can provide consent. The Wechsler Adult Intelligence Scale IV will be used to evaluate general cognitive ability (intelligence quotient) for all subjects at baseline.
3. Assessments of changes in working memory, other cognition and function (primary outcomes) will be administered repeatedly at baseline, 1 month after training (either adaptive working memory or ‘active control’ training condition), and 6 months after the adaptive working memory training. Alternative word lists or stories will be used for repeated testing. Table 2 shows the tests involved in the trained working memory tests, which will include the ‘start index, maximum index, and improvement index’, the non-trained working memory tests and the transfer of working memory capacity gain to other cognitive functions.

For the non-trained working memory tasks, no practice effects are expected [64]. However, for the other neuropsychological tests, variable degrees of practice effects will occur with test–retest [65]; we will control for such practice effects by performing the same tests before and after CWMT in both HIV-positive and seronegative control subjects.

### Cognitive intervention using Cogmed® RM

The Cogmed® RM (Table 3) was developed for school-aged children; however, we chose this version for our adult participants since it is more colorful and appealing to the participants than the adult version (Cogmed® QM). The training program consists of 25 training sessions (minimum of 20 required), 30–40 minutes per day, 4–5 days per week over a 5–8 week period [35]. Subjects will perform either the ‘active control’, with a fixed low level across each task, or an ‘adaptive’ version, with increasing difficulty for the working memory tasks. The training will be performed either in the participants’ homes, or at the research laboratory. We will ensure that participants have high-speed internet access. Subjects will be trained using verbal and non-verbal working memory tasks that involve: (1) maintenance of several stimuli at the same time; (2) short delays during which the representation of stimuli should be held in the working memory; (3) unique sequencing of stimulus order in each trial. Training will be supplemented with weekly guidance by trained ‘coaches’, who can review subjects’ progress online and will telephone them to give feedback, motivation, and advice to optimize their training experience.

**Table 3:** Description of the 12 modules in Cogmed® Working Memory Training

Task	Description
Verbal working memory (with visuospatial components)	
Input module (numbers)	A panel with 1 to 9 is shown. Numbers are presented aurally and light up on the panel sequentially. Participants then have to click on the numbers in the reversed sequence.
Input module with lid (hidden numbers)	Same as previous task, except numbers are presented aurally only, since they are covered by a lid, which opens when all the numbers are read.
Stabilizer (letters)	A circle of lamps is shown. A sequence of letters is presented aurally along with specific lamps that light up. Participants are then presented with a letter in the middle of the circle, and have to click on the lamp that lit up.
Decoder	Letters are read sequentially along with a lamp that lights up. The participant then has to reproduce the sequence by selecting one letter from three possible letters below each lamp.
Visuospatial working memory	
Visual data link (grid)	Lamps light up sequentially in a 4 × 4 grid, and the participant must reproduce the sequence by clicking on the lamps in the same order afterwards.
Rotating data link (rotating grid)	Same as the grid task above, except that after the lamps light up, the entire board rotates 90° and the participant has to click on the sequence of lamps in the rotated grid.
Data room (three-dimensional grid)	Lamps will light up sequentially in a three-dimensional box (view from the front), with four lamps per plane, and the participant must reproduce the sequence by clicking on the lamps.
Three-dimensional cube	The program zooms in on different planes of a cube (four squares on the back and two squares on the other planes) as they light up sequentially. The participant must click the squares in the correct order.
Rotating (rotating dot)	Lamps light up sequentially on a circle, which rotates slowly clockwise, and the participant has to reproduce the sequence by clicking on the lamps on the moving circle.
Sorter	Boxes open to reveal numbers out of order and the participant must click on the boxes in the correct numerical order.
Asteroids	Asteroids light up sequentially while moving around the screen and the participant must reproduce the sequence by clicking on the moving asteroids.
Space whack	Craters emit puffs of smoke sequentially. The participant must remember the order that the puffs appeared, in order to click on the alien that will pop out of the crater in the same sequence.

### Participant randomization

Individuals meeting all study criteria will be randomized, with 30 participants assigned to each training condition to maintain a balanced design. Randomization to study condition will be stratified across characteristics that might affect outcomes. Stratification variables include age, sex (male, female), and severity of cognitive deficits (based on the global cognitive z-score). This random assignment will ensure that both the participant and the ‘coaches’ (who will monitor the subjects online and by phone) for the training will be blinded to the training being used. No other members of the study team (except for the principal investigator) or participants will be informed of which condition assignment has been made. The randomization assignment will remain blinded until 1 month after the training and the follow-up visit are complete. Subjects who were trained on the active control (low-level training) condition will then be offered the opportunity to be trained on the intensive adaptive working memory training (open-label phase), and will be followed-up again at 1 month and 6 months after the adaptive training. Subjects unable to participate in the open-label phase during this period will be given the option to continue participation after the active control 6-month follow-up. The data manager will maintain the records that link specific participant identification numbers to study conditions, and assignment codes. This information will be available for the principal investigator in case there is any need to break the study blind for a specific participant while the individual is still in the study.

### MRI

All participants will undergo a series of MRI sessions before, and 1-month and 6-months after CWMT. All structural and fMRI studies will be performed on a research-dedicated 3 Tesla Siemens TIM Trio scanner (VB17), using a 12-channel phase-array head coil.

#### Structural MRI

A 3D magnetization-prepared rapid gradient-echo scan will be performed (sagittal, TE/TR/TI = 4.9 ms/2.2 s/1 s, 7° flip angle, 1 mm resolution covering the whole-brain). Next, a 3D-T2-weighted sequence (SPACE) will be acquired (sagittal, TR/TE = 3200/447 ms, field of view = 320 mm, 1.0 × 1.0 × 1.0 mm resolution, 192 slices, generalized autocalibrating partially parallel acquisitions (GRAPPA) = 2, 3:52 min). Both scans will be reviewed to screen for possible brain lesions or structural abnormalities.

#### BOLD-fMRI

The entire brain is scanned continuously with an axial single-shot gradient-echo echo planar imaging sequence (TE/TR30/2500 ms, 20 cm field of view, 64 × 64 resolution, ≈35 × 3 mm slices, no gap) with motion correction. An electronic pulse from the scanner synchronizes the fMRI scan and stimulus presentation. Scans with excess motion (more than ±1 mm translations or ±1° rotations) are repeated immediately*.*

#### fMRI paradigms

Each subject will perform six different tasks during the fMRI scans. Tasks will be displayed using a binocular video display (Nordic Neurolab Inc.™). Subjects will be trained first outside the scanner to ensure that they will be able to perform the tasks well during the scans (>80 % accuracy, monitored during the scan). Scans with unacceptable response accuracy will be repeated immediately.

#### Working memory (n-back)

This task uses a block design (30 s of alternating control and activation periods, including 3 s of instruction). During activation periods, a random sequence of single letters is presented (1 letter per second for 500 ms). Subjects are trained to press a button whenever the current letter is identical to the letter 1 or 2 events back, or for any number (0-back). The targets (i.e., 0-, 1-, or 2-back events) are random events (5/30 s block). Button events are used to determine accuracy and reaction times. During control periods, subjects passively view a random sequence of symbols (non-letters), with matching font size, brightness, and timing.

#### Visual attention (ball tracking)

These tasks evaluate non-verbal visual attention [66, 67], which we also expect to improve after working memory training. Subjects mentally track two, three, or four target balls that are highlighted briefly among 10 randomly moving, colliding balls. Every 12.5 s, another set of balls is highlighted for 1 s and the subjects push a button if subjects agreed that the balls were the original targets (performance monitoring). Periods of tracking (1 min) alternate with passive viewing (1 min) of balls that move in the same random motion without highlighting (block design: 1 min/period × 2 periods × 3 blocks).

### Genotyping

Samples of DNA will be extracted from whole blood or saliva collected in EDTA-tubes using DNeasy Blood & Tissue Kit (catalog number 69506, Qiagen Inc., Valencia, CA, USA). Genomic DNA will be subjected to restriction fragment length polymorphism PCR to genotype each participant for *LMX1A* at rs4657412 as described previously [68]. Specifically, approximately 3ng of genomic DNA will be amplified for LMX1A using the primer LMX-5': 5'-CTCGCCTCCAGGAATGGGTGTTGTA-3' and LMX-3': 5'-GCCACGAGGAACTTGTGAGAGGGTT-3', and under the following conditions: denaturation at 94C for 5 min, followed by 30 cycles at 94 °C for 30 sec, annealing at 64 °C for 30 sec, and extending at 72 °C for 30 sec. 15 ul of the amplification products will be digested by 2.5U of MslI (R0571s, New England Biolabs, Beverly, MA) for 2 h at 37°C. The digested  PCR products will be then analyzed on a 4 % agarose gel and visualized using GelGreen® Nucleic Acid Gel Stain (89139-144, Biotium, Hayward, CA).

### Cerebrospinal fluid cytokines/chemokines and dopamine/dopamine-related metabolite measurements

Lumbar punctures will be performed at baseline and 1 month after CWMT in participants who have agreed to spinal taps. The cerebrospinal fluid will be collected, aliquoted into 0.5 ml portions, and swiftly frozen at −80 °C. Measurements for both ELISAs and monoamines will be run in batches.

#### ELISA

We will quantify fractalkine, IL-1α, MCP-1, IP-10, IL-8, and IL-4 using the RayBio ELISA kits (RayBiotech, Inc., Norcross, GA, USA). Since fractalkine concentration is relatively low even in normal serum and plasma, and may not be detected in the RayBio assay, we plan to use the Human Fractalkine ELISA kit (ADIPO Bioscience, Inc., Santa Clara, CA, USA), which is reportedly more sensitive [69].

#### Cerebrospinal fluid dopamine and dopamine metabolite levels

These will be measured using HPLC assays [70] with electrochemical detectors and using principal component analysis precipitation to remove proteins and stabilize small molecule analytes. Internal standards will be used to measure monoamines and their metabolites, including norepinephrine, dopamine and its metabolites, 3,4-dihydroxyphenylacetic acid and homovanillic acid, as well as serotonin (5-hydroxytryptamine) and its metabolite, 5-hydroxyindole-3-acetic acid.

### Statistical analysis

All analyses will be performed using SAS, using repeated-measures analysis of variance (ANOVA) as the primary model (co-varied for age and sex). The baseline (initial) values of all key variables will be tested for normality prior to analysis. Appropriate transformations (or non-parametric tests) will be used as necessary. The statistical significance will be determined using a modified Bonferroni procedure for multiple tests [71]. The procedure will have a type I error probability equal to 0.05 for independent tests.

Neurocognitive assessments will be tested using appropriately constructed contrasts within a 2 × 2 repeated-measures ANOVA model. HIV status (and HAND status for subgroup analyses) and training condition (adaptive versus fixed) will be across-subjects variables, and time (baseline, 1-month, 6-months) will be within-subject variables. Analyses will be co-varied for subject age, sex, and other co-variates as needed (e.g., depression scores). Subjects trained in both the adaptive and fixed training will have the training condition as a within-subject variable.

The fMRI time series will be analyzed using Statistical Parametric Mapping software (SPM8, Welcome Department of Cognitive Neurology, UK). After spatial normalization and smoothing, maps of brain activation and differential changes in activation (repeat − baseline) will be calculated for each subject and task, using a fixed-effects model with a block design. In a subsequent random-effects analysis, differences between groups on BOLD signal changes will be evaluated for each task (*t* tests or ANOVA). Statistical significance will be based on cluster-level significance at *P* < 0.05 corrected for multiple comparisons (voxel-level threshold *P* < 0.05, cluster size >100 voxels). Additionally, we will extract regional percentage BOLD-fMRI signals from cubic regions of interest (0.729 cm^3^) centered at cluster maxima, using a customized program written in Matlab. The extracted data will be imported into SAS, where a repeated-measures ANOVA model will be run to confirm and further evaluate the statistical parametric mapping findings.

For the genetic study, we will first determine the Hardy–Weinberg equilibrium for both allelic and genotype frequencies of the *LXM1A* gene in our cohort. To minimize the number of correlations, we will use repeated-measures ANOVA to select cognitive variables and extracted fMRI data that show changes from baseline to 6 months after CWMT (adaptive versus active control). We will assess the relationship of these variables with the *LMX1A* genotypes and with cerebrospinal fluid markers using ANCOVA. Repeated measure ANCOVAs and multivariate ANCOVAs will also be utilized.

## Dissemination plan

At the completion of this trial, we plan to submit several manuscripts to peer-reviewed journals including;

1. ‘Neural correlates of adaptive working memory training in HIV patients’: this paper will report on the interim findings from the primary outcome measures, including improvements on working memory performance and neural efficiencies, 1 month and 6 months after the adaptive version of CWMT, to assess the gain and maintenance of working memory improvements after training. Some of the secondary contrast results, including scoring on the Behavior Rating Inventory of Executive Function–Adult Version and *LMX1A* genotype effects, will also be reported. These findings may lead to a larger study to validate whether CWMT can be used as an adjunctive therapy for HAND.
2. ‘Computerized working memory training in HIV patients: a double-blind active control study’: this paper will be written at the completion of subject enrollments. We will compare the effects of adaptive versus fixed low level of CWMT on the primary outcome measures, at 1 month and 6 months after training compared with baseline assessments. This will provide further evidence of whether CWMT is effective for working memory and attention deficits in HIV patients.
3. This paper will also report on the findings after the double-blind active control study to evaluate the efficacy of CWMT on the secondary outcome measures, including scores on the the Center for Epidemiological Studies–Depression Scale, Revised, and the Symptom Checklist–90–revised, and ability to conduct activities of daily living (Independent Activity of Daily Living assessment and the Behavior Rating Inventory of Executive Function–Adult Version) before and after CWMT.
4. This paper will report on possible improvements in cerebrospinal fluid neuroinflammatory proteins (chemokines and cytokines) and neurochemicals (dopamine and serotonergic levels and their metabolites) after CWMT. Since only half of the participants might consent to the lumbar punctures, we may need to combine results from the adaptive training group and the fix-low-level training group to evaluate the training effects on these cerebrospinal fluid measurements.

We plan to submit our first manuscript on the interim findings before the end of the project in 2015, and the other three papers in 2016 or 2017. The findings will also be presented and discussed at international conferences and workshops, as well as at local symposia to our community healthcare providers.

## Discussion

Cogmed Working Memory Training® may be a useful adjunctive treatment for HAND in HIV-positive patients who are receiving combined antiretroviral therapy. Since CWMT is non-invasive and can be accessed from any computer via the internet, this safe intervention is easily accessible and costs less than typical pharmacological treatments. Furthermore, the proposed work may provide direct and immediate novel data on the long-term maintenance effect of adaptive working memory training, and insight into how the brain and the neural networks involved in working memory and attention may be modified by the training.

The proposed research has several innovative aspects. (1) We will evaluate a new, non-invasive approach to improve cognitive function in HIV-positive subjects. Except for a single recent report of a pilot study of ‘internet-based cognitive stimulation’ (using SmartBrain©) [72], the proposed intervention with CWMT is, to our knowledge, the first to evaluate whether working memory and other cognitive domains can be improved in HIV patients. (2) This will, to our knowledge, be the first study to assess neural plasticity of cognitive training in HIV patients. We will evaluate the neural correlates of the cognitive improvements associated with the intervention, using fMRI techniques. (3) We will genotype subjects to determine whether individuals with the TT(AA) alleles for the *LXMI1A* gene perform better at working memory training. This may pave the way for personalized treatments in the future. (4) Further insights will be derived from studying the relationships between neuroinflammation and brain function (correlations among neuropsychological test scores, working memory performance from the CWMT program, and cerebrospinal fluid cytokines or chemokines). Our approach will provide unique insights into how the brain changes after cognitive training. If successful, the study could lead to a larger intervention study to determine whether working memory training may prevent or improve behavioral and cognitive problems that affect HIV-infected patients. Training the brain might be analogous to physical exercise to prevent cardiovascular diseases, and lead to a new era of prevention for brain disorders.

### Expected findings

#### Gain in working memory performance and transfer of gain to ‘near’ or ‘far’ transfer tasks (Aim 1)

HIV-positive subjects commonly have working memory deficits [3, 23–29, 41–43]. Since working memory is needed to maintain focus on tasks, deficits in working memory may negatively affect many other cognitive domains, and result in HAND. Prior studies of CWMT showed that the program enhanced performance not only on trained working memory tasks, but also on some non-trained visuospatial and non-trained complex reasoning tasks, including executive function [73]. Therefore, we aim to evaluate the efficacy of CWMT on cognitive performance in our subjects.

We hypothesize that all subjects will show improved working memory after Cogmed® RM training (adaptive training > low fixed level ‘active control’ training), both at 1 month and at 6 months after cessation of training. In addition, not only will adaptive CWMT will lead to greater improvements than active control training on the trained tasks, but the gain will transfer to non-trained tasks that require similar working memory processes, including near-transfer tasks (e.g., digit span and spatial span) and far-transfer tasks (e.g., verbal learning, logical memory, short-term memory, and recognition memory). Lastly, we expect that the gain will be greater in subjects with normal cognition (regardless of HIV status) than in those with cognitive deficits (e.g., HAND or HAND-equivalent).

#### BOLD-fMRI studies (Aim 2)

BOLD-fMRI has been used to evaluate brain changes in HIV-positive patients for more than a decade. BOLD-fMRI signals in these individuals are altered during tasks that require working memory [74, 75] and visual attention [74, 76]. A prior study showed that HIV patients had a lower working memory network capacity, which was further affected by other concurrent brain activities (e.g., louder acoustic noise proportionally decreased working memory activation); hence, they would be distracted more easily by other activities in their daily life [75]. Hence, we expect that subjects with normal cognition (higher working memory capacity) will show greater improvement than those with cognitive deficits.

BOLD-fMRI signals during working memory tasks were also greater in cognitively normal HIV-positive subjects compared with seronegative controls [77], as well as in HIV-positive subjects receiving antiretroviral medications compared with both HIV-negative and antiretroviral medication-naïve subjects and seronegative controls [6]. Greater BOLD-fMRI signals in HIV patients were predicted by higher levels of glial metabolites (myoinositol, total creatine, and choline) [78]. These findings suggest that ongoing increased neuroinflammation in HIV patients necessitates greater usage of the working memory network. Such neuroinflammation appeared to be ongoing despite stable antiretroviral treatments, since HIV-positive subjects showed increased BOLD-fMRI signals within the visual attention network in a one-year longitudinal follow-up study (suggesting decline efficiency), while matched seronegative controls showed decreased BOLD-fMRI signals (consistent with a test–retest practice effect) [79]. Therefore, this study will also explore how neuroinflammation as assessed by cerebrospinal fluid inflammatory markers is related to BOLD-fMRI signals before and after working memory training.

For this aim, we predict that all subjects will show improved BOLD signals with decreased activation, indicating greater neural efficiency, in brain networks associated with working memory (*n*-back) and visual attention (ball tracking), at 1 month and 6 months after adaptive working memory training compared with baseline and relative to active control training. Also, brain activation will normalize in HIV-positive subjects, who will show smaller group differences compared with seronegative controls at 1 month and even smaller differences at 6 months after adaptive working memory training.

#### Genotype effects (Aim 3)

Since HIV patients have dopaminergic deficits, with decreased dopamine transporters [18, 19] and decreased cerebrospinal fluid dopamine and dopamine metabolites [62], genetic variations in genes related to dopamine metabolism, such as *LMX1A* or catechol-O-methyltransferase, may modulate working memory training effects, BOLD-fMRI results, and cerebrospinal fluid monoamine levels [55]. For instance, while HIV-positive subjects with the Met/Met alleles at the catechol-O-methyltransferase *Val58Met* gene (a gene involved in the degradation of released dopamine) perform better on executive function [80], healthy individuals with the TT(AA) alleles at the *LMX1A*-rs4657412 benefit more from working memory training than those individuals with the C(G)-carrier [55]. Therefore, we predict that polymorphism of the *LMX1A* gene will differentially affect cognitive improvements among HIV-positive individuals. Specifically, consistent with a prior report, we expect that, regardless of HIV serostatus, subjects with the TT(AA)-alleles at the *LMX1A*-rs4657412 will perform better on verbal working memory tasks and have greater training-related gains in verbal working memory than those carrying the C(G)-allele after CWMT [55], especially in subjects with normal cognitive function. We also expect that TT(AA)-carriers will show greater improved neural efficiencies, i.e., greater decreases in BOLD-fMRI, than TC(AG) or CC(GG) carriers.

#### Cerebrospinal fluid neuroinflammatory markers and monoamine metabolites (exploratory aim)

Numerous studies indicate that ongoing aberrant neuroinflammation in HIV-infected individuals might contribute to brain injury associated with HAND [4, 81–83]. In prior studies, cerebrospinal fluid level of MCP-1 correlated with brain metabolites [84], and the three cerebrospinal fluid chemokines (MCP-1, IP-10, and IL-8) had the strongest correlations with cerebral metabolite patterns [82]. We will measure pro-inflammatory cytokines (fractalkine, INF-α2, and IL-1α), chemo-attractants (MCP-1, IP-10, and IL-8) and anti-inflammatory cytokines (IL-4) in the cerebrospinal fluid of our study participants. We hypothesize that, at baseline, levels of pro-inflammatory cytokines (fractalkine, INF-α2, and IL-1α) and chemo-attractants (MCP-1, IP-10, and IL-8) will be higher, while the anti-inflammatory cytokine (IL-4) will be lower in HIV-positive subjects, especially in those with HAND, compared with seronegative controls. These markers will correlate with cognitive performance and BOLD-fMRI signals at baseline more so than at 6 months after training.

Furthermore, many symptoms of HAND are consistent with mild Parkinson’s disease; correspondingly, HIV patients show dopaminergic terminal deficits, with decreased dopamine transporters [18, 19], and decreased cerebrospinal fluid dopamine and dopamine metabolites [62]. Dopamine transporters also correlated with deactivation in the default mode network on visual attention task [85]. Furthermore, changes in cortical dopamine D1 receptor binding observed by positron emission tomography were associated with improvement from CWMT [86]. Therefore, we expect cerebrospinal fluid monoamine and metabolite levels to be lower in HIV than in seronegative subjects at baseline, but levels may increase after CWMT. We also expect that these levels will correlate with cognitive function and BOLD-fMRI signals both before and after CWMT.

### Additional considerations and future directions

1. Based on our pilot study, individuals with HAND had the highest dropout rate, probably owing to frustrations or lack of motivation. Therefore, we will spend more time to train these participants in our laboratory initially and coach them during the early phase of the training (contacting them three or more times per week).
2. We will also ensure that each participant will have a computer and high-speed internet access (e.g., by providing internet cards and inexpensive computers, or by training them in our laboratory, which we have done successfully during the pilot study). The training program, CWMT, can be accessed via internet from any computer.
3. Furthermore, while typical medication trials terminate clinical contact after the medication phase, we plan to follow the subjects for 6 months after CWMT; this allows us to collect novel data on the long-term maintenance effect of adaptive working memory training and determine whether there are any carry-over (positive or negative) effects of the experimental condition.
4. Lastly, we will collect information regarding physical exercise and sleep patterns, which could either enhance or interfere with both baseline cognitive functioning and their training outcomes [87–89]. As discussed, cognitive training or stimulation might be analogous to physical exercise to prevent cardiovascular diseases, and lead to prevention or delay development of dementia both in seronegative and HIV-positive subjects.

The proposed research has significant public health impact since HAND is an increasing problem with significant impact on the lives of afflicted patients and a growing financial burden to society. The findings from this protocol may provide foundational information necessary for the development of a potential adjunctive non-pharmacological treatment for HAND.

HIV-positive patients with HAND experience difficulties in activities of daily living and negative emotional well-being. Cognitive deficits and impairment of working memory are often overlooked, but may in fact be ‘treatable’. The data reviewed clearly demonstrate the urgency of this emerging problem, especially as the HIV-infected population continues to grow and age. Therefore, our proposed research has high significance in that the findings may lead to an intervention that might improve the cognitive function of HIV-infected individuals with HAND, and possibly delay or prevent the development of HAND in others.

### Trial status

The trial started in September 2012, and is currently enrolling participants.

## Abbreviations

ANCOVA: analysis of covariance
ANOVA: (analysis of variance)
BOLD: blood-oxygenation level-dependent
CWMT: Cogmed Working Memory Training®
ELISA: enzyme-linked immunosorbent assay
fMRI: functional magnetic resonance imaging
GRAPPA: generalized autocalibrating partially parallel acquisitions
HAND: HIV-associated neurocognitive disorders
HIV: human immunodeficiency virus
HPLC: high performance liquid chromatography
IL: interleukin
LMX1A: LIM homeobox transcription factor 1, alpha
MRI: magnetic resonance imaging
PCR: polymerase chain reaction
TE: echo time
TI: inversion time
TR: repetition time.
